# A key bacterial cytoskeletal cell division protein FtsZ as a novel therapeutic antibacterial drug target

**DOI:** 10.17305/bjbms.2020.4597

**Published:** 2020-08

**Authors:** Mujeeb ur Rahman, Ping Wang, Na Wang, Yaodong Chen

**Affiliations:** 1 Key Laboratory of Resources Biology and Biotechnology in Western China, Ministry of Education, College of Life Sciences, Northwest University, Xi’an, China; 2 Department of Anesthesiology, Duke University Medical Center, Durham, North Carolina, USA

**Keywords:** FtsZ, multidrug resistance, cell division, antimicrobial, inhibitors, natural antimicrobial compounds

## Abstract

Nowadays, the emergence of multidrug-resistant bacterial strains initiates the urgent need for the elucidation of the new drug targets for the discovery of antimicrobial drugs. Filamenting temperature-sensitive mutant Z (FtsZ), a eukaryotic tubulin homolog, is a GTP-dependent prokaryotic cytoskeletal protein and is conserved among most bacterial strains. *In vitro* studies revealed that FtsZ self-assembles into dynamic protofilaments or bundles and forms a dynamic Z-ring at the center of the cell *in vivo*, leading to septation and consequent cell division. Speculations on the ability of FtsZ in the blockage of cell division make FtsZ a highly attractive target for developing novel antibiotics. Researchers have been working on synthetic molecules and natural products as inhibitors of FtsZ. Accumulating data suggest that FtsZ may provide the platform for the development of novel antibiotics. In this review, we summarize recent advances in the properties of FtsZ protein and bacterial cell division, as well as in the development of FtsZ inhibitors.

## INTRODUCTION

The antibacterial resistance phenomenon has seen an increase to dangerous, unprecedented levels in practically all parts of the world over the past 20 years. While antibiotic resistance was once generally restricted to the hospitals and long-term care facilities, it has been emerging now in community settings, becoming one of the world’s most pressing concerns associated with the topic of public health [1]. In 2017, the Center for Disease Control (CDC) estimated 19,832 methicillin-resistant *Staphylococcus aureus* (MRSA) related deaths in the United States [2]. Based on the extent of threat, CDC categorizes these threats into three levels: “concerning” indicates the lowest level of threat, “serious” classifies as optimum level, while “urgent” falls in the highest level of resistance. As fatal diseases that rank as “serious” on the threat level, immediate action is required to ensure that the threats of MRSA and extensively drug-resistant and multidrug-resistant *Mycobacterium tuberculosis* (XDR/MDR-TB) do not upgrade to the highest level. According to the CDC, the urgent threats include carbapenem-resistant *Acinetobacter*, *Candida auris*, *Clostridioides difficile*, carbapenem-resistant Enterobacteriaceae (CRE), and drug-resistant *Neisseria gonorrhoeae* [3]. Not only pathogenic strains of bacteria have increased the multidrug resistance to antibiotic, but there is also a lack of advancement in the discovery of new antibiotics agents; hence, they have punctuated the need for novel chemotherapeutic strategies [4]. Conventionally, the cellular processes of transcription, folate biosynthesis, translation, cytoplasmic membrane, peptidoglycan biosynthesis, DNA replication, and bacterial structures among others were targeted by all known clinical antibiotics [5]. Now, urgency is placed on exploring new molecular targets and new strategies for antibiotic development; one of the most promising targets to develop novel antibiotics is bacterial cell division.

Cell division, or cytokinesis, is the essential process for the survival of all bacterial cells [6,7]. It is a three-step process in rod-shaped bacteria, comprising first of cell elongation, then septum formation, and finally cell division into two identical daughter cells. Filamenting temperature-sensitive mutant Z (FtsZ) is a key cytoskeletal cell division protein in most bacteria. At the division site, during the process of cell division, FtsZ forms ring-like structure (Z-ring), takes on the role as a scaffold for the recruitment of multiprotein complex (known as divisome), and may also generate the force that is vital for the viability of the cell [8-10]. The Z-ring, besides being very dynamic *in vivo* [11,12] as well as consisting of mainly FtsZ and dozens of other proteins, is also known to take part in the localization and stability of divisional proteins through a considerable number of protein-protein interactions [13]. Over the past 30 years, the bacterial cell division has been a substantial topic of discussion for the development of novel antimicrobials compounds [14,15].

The current review aims to discuss the biochemical properties of FtsZ proteins and assess what makes them an appropriate target for antibacterial drugs. Furthermore, we discuss the example of compounds that have been identified to interrupt FtsZ assembly and its interaction with other proteins. Correspondingly, we deliberate the significant challenges in developing drugs against this novel target.

## PATHWAY OF BACTERIAL CYTOKINESIS

The process of cytokinesis has been widely studied in *Bacillus subtilis* and *Escherichia coli*, as well as in *S. aureus* [16-20], *Streptococcus pneumonia* [21-24], and *M. tuberculosis* [25-32]. In these bacterial cells, the cell envelope layer is invaginated by the divisome which, in turn, forms a septum between replicated chromosomes at the middle of the cell. Dozens of proteins recruited to the site of divisome at the cell center are involved in the process of septation. FtsZ, a bacterial tubulin homologue, is the center of the divisome (contractile ring, Z-ring). Many of these proteins in *E. coli* and *B. subtilis*, named filamentous temperature-sensitive (Fts), have been determined by screening for a temperature-sensitive mutant that grows normally and divides at the permissive temperature, but at the restrictive temperature, it continuously elongates without the division of cell. However, some proteins are also used, such as Div that stands for the division in *B. subtilis*, and are recognized through other methods [33]. More than two dozen proteins have been found as components or regulators of the divisome, and the number is still increasing. There might be different associated proteins in different bacteria. In *E. coli*, an abundance of proteins is essential for cell division, including FtsZ, FtsA, ZipA, FtsE, FtsX, FtsK, FtsB, FtsQ, FtsL, FtsW, FtsN, and FtsI. Studies revealed that *E. coli* has a consecutive, almost straight assembly of the protein’s pathway in the following direction: FtsZ [FtsA, ZapA, ZipA] (FtsE, FtsX), FtsK, FtsQ, (Fts B, FtsL), FtsW, FtsI, FtsN, AmiC, and EnvC [34-39].

At the early stage of the Z-ring assembly, FtsZ polymers anchor to the membrane by FtsA and ZipA at the future division site *in E. coli*. However, in *B. subtilis*, FtsA, ZapA, SepF, and perhaps EzrA are first localized to Z-ring of FtsZ proteins. Moreover, these are dependent on FtsZ for their localization to the site of cell division. The downstream proteins such as DivIC (FtsB from *E. coli*), DivIB (FtsQ from *E. coli*), FtsL, PBP2B (FtsI from *E. coli*), and possibly FtsW are recruited next for the localization of septum [31]. Although new studies of *E. coli* have shown that instead of arising as a sequence of binary interactions, the divisome assembly occurs as a complex network of protein-protein interactions [40], it is proposed that in these two organisms the assembly of the divisome complex could potentially be more parallel than once supposed.

FtsZ STRUCTURE AND FUNCTION

FtsZ is a structural homolog of eukaryotic cytoskeletal protein tubulin [41,42]. The sequence similarity of FtsZ in the majority of archaeal and bacterial species is about 40–50% [43]. Although the sequence resemblance between FtsZ and tubulin is <20%, they have a high structural identity [44]. In a single *E. coli*, there are approximately 5000 copies of FtsZ [45]. FtsZ polymerizes into protofilaments, and at the center of the dividing cell, they are aligned to form the Z-ring [10].

## TREADMILLING DYNAMICS

Like eukaryotic tubulin, FtsZ binds and hydrolyzes guanosine-5’-triphosphate (GTP); on the other hand, in the presence of GTP, it forms a single-stranded protofilament or bundles instead of forming hollow microtubules. FtsZ from *E. coli* assembles into mostly single protofilaments that are highly dynamic, with an average of 200 nm in the presence of GTP *in vitro* [46]. The filaments will disassemble after GTP hydrolysis with a halftime of the subunits exchange of around 5–7 seconds [46-48], exhibiting a dynamic treadmilling action [49]. It is very consistent with the halftime of the exchange rate of the Z-ring *in vivo*, which is around 8–10 seconds [12]. Two studies in 2017 showed that FtsZ protofilaments demonstrate polarity and undergo treadmilling in both *B. subtilis* and *E. coli* cells [50,51]. During treadmilling process, subunits are selectively bound to one end and detached from the other end, and as a result, single FtsZ protofilaments or bundles travel as a unit around the cell circumference, following the irregular path of the ring. Although each treadmilling filament moment is processive, in the same cell, different FtsZ protofilaments can move in the opposite direction. Thus, it appears that each unit of protofilament has its own directionality [7,52]. Occurring inside protofilaments or by treadmilling at the ends of protofilaments, the turnover in the FtsZ subunit, regulated by FtsZ GTPase activity, is proportional to treadmilling [53]. It remains uncertain how single-stranded FtsZ protofilaments treadmill as they have innate polarity, and why one end gains while the other loses subunits. Wagstaff et al. suggested a possible mechanism; by examining the crystal structures of FtsZ, a correlation was identified between various FtsZ conformational states and its abilities to form polymers [18]. As treadmilling and polymerization of FtsZ are crucial for normal septal morphology, the inhibition of such activities should inhibit the process of cell division.

Recently, Monteiro et al. studied the effect of the compound PC190723, an FtsZ inhibitor, and DMPI (3-{1-[(2,3-dimethylphenyl) methyl]piperidin-4-yl}-1-methyl-2-pyridin-4-yl-1*H*-indole), the lipid II flippase MurJ inhibitor, on the Z-ring of *S. aureus* cells [54]. It has been demonstrated that the PC190723 compound inhibits FtsZ treadmilling and blocks the constriction of Z-ring only at initial stages, whereas the MurJ inhibitor DMPI has been shown to block the ring constriction at all stages. Furthermore, many small molecules and peptides, either synthetic or natural, target FtsZ polymerization, some having the potential to initiate antimicrobial activities. Based on the research that treadmilling motion of the FtsZ protein is essential for cell division, it may be possible to develop a novel drug that precisely prevents this motion, similar to how the chemotherapy drug taxol suppresses the moment of the cytoskeleton in malignant growth cells [55].

## FTSZ AS A NOVEL ANTIBACTERIAL DRUG TARGET

Antibiotics are one of the most prominent weapons to use against microbial infections [56]. However, currently, the misuse of antibiotics creates selective pressure for the existence of resistant bacterial strains, and subsequently, several clinically used antibiotics, such as aminoglycosides, β-lactams, sulfonamides, tetracyclines, are becoming less effective against antibiotic-resistant bacteria [57]. Therefore, to control these problems, finding novel medicines is a necessity. Bacterial cell division proteins have several features; for this reason, they are suggested to be the right candidate for the antibacterial goal. First, FtsZ is a key protein for the viability of bacterial cells [58-60]. In addition, the cell division proteins in most of the bacteria are highly conserved but mainly lack or have limited sequence similarity in eukaryotic cells. The third benefit is that FtsZ is absent in higher eukaryotes, which asserts that inhibitors of FtsZ will not be toxic to human cells [41,61,62]. During the last few decades, the biological and biochemical properties of FtsZ caught the attention of scientists. A biological molecule that inhibits the GTPase activity or assembly of FtsZ can establish an active antibacterial agent.

Due to the structural similarity between FtsZ and tubulin, the tubulin inhibitors and their derivation could be used to screen anti-FtsZ compounds. Scientists at the Southern Research Institute screened the library of 200 2-alkoxycarbonylamin-o-pyridine compounds, which are inhibitors of tubulin polymerization, to inhibit FtsZ and control *M. tuberculosis* growth. With this approach, they found many compounds that have desirable properties [63]. Colchicine, a well-recognized tubulin inhibitor, along with the compound 2-alkoxycarbonylamin-o-pyridine SRI-3072 and SRI-7614, inhibited the polymerization and GTP hydrolysis of *M. tuberculosis* FtsZ in a dependent manner. Both components (SRI-3072 and SRI-7614) were effective against both drug-resistant and susceptible strain of *M. tuberculosis*. SRI-7614 inhibited both tubulin and FtsZ, while SRI-3072 only inhibited FtsZ polymerization. In addition, they showed that SRI-3072 could inhibit *M. tuberculosis* growth in the infected bone marrow macrophages of mice and was able to effectively enter intracellular parasite and even the host cell [29,63].

Even though FtsZ and tubulin show high structural similarity, there are some structural features of FtsZ that are different from tubulin [62]. First, the primary sequence of FtsZ differs from that of tubulin by more than 80% [53]. Second, all the subunits of FtsZ are similar, while the microtubules consist of two types of tubulin subunits (α and β). In addition, the lateral interaction of FtsZ protofilament changes from the longitudinal association of tubulin protofilaments, which leads to the development of an arch-shaped Z-ring [64]. These evidences are useful for the development of new antibacterial agents that will ultimately target the FtsZ but with few side effects. Actually, most of the tubulin inhibitors such as albendazole, nocodazole, thiabendazole, 3-methoxybenzamide, or paclitaxel show little inhibition of FtsZ polymerization or GTPase activity *in vitro* [65]. It is demonstrated that such an inhibitor can be determined to discriminate between FtsZ and tubulin.

## NATURAL-PRODUCT SCREENING FOR FtsZ

The natural sources of medication have a key role in the treatment of human diseases. These traditional medicines have become an integral part of the primary healthcare organization [66]. It is reported that herbal plants have tremendous healing potentials and play a crucial role in traditional medicines [67]. Here, we describe natural products and their derivatives, which showed significant inhibition on FtsZ and divisome.

### Alkaloids

Sanguinarine, known as the bloodroots, is a benzophenanthridine alkaloid derived from the rhizomes of *Sanguinaria canadensis*, having a wide variety of antimicrobial activities. In both *E. coli* and *B. subtilis*, it causes filamentation, which indicates a cell division defect. It is proposed that sanguinarine is not limited to inhibiting FtsZ assembly; it can also reduce FtsZ bundling by directly binding FtsZ. The cytotoxic ability of sanguinarine can be inferred due to this compound’s ability to depolymerizing microtubules [68,69].

Berberine is a planted alkaloid extracted from many species of *Berberis*. It has been reported that berberine inhibits *E. coli* FtsZ assembly and its GTPase activity *in vitro* [70]. It is predicted that the berberine binding spot overlaps with the GTP binding pocket in *E. coli* FtsZ. Given the availability of crystal structure of *S. aureus* FtsZ, a series of 9-phenoxyalkyl berberine derivatives were designed that could attach to the *S. aureus* FtsZ interdomain cleft. The value of minimum inhibitory concentration (MIC) was 2–8 µg/mL against *S. aureus* and 4–16 µg/mL against *Enterococcus faecalis*. Furthermore, the berberine analogs prevent the growth of Gram-negative organisms such as *Klebsiella pneumoniae* and *E. coli* with 32–128 µg/mL minimum inhibitory concentration (MIC) values [57]. Therefore, further study is needed to ascertain the antimicrobial action of berberine.

### Phenylpropanoids and terpenoids

Phenylpropanoids are secondary metabolites of the plant that protect plants from predators and pathogens. Many of these compounds have antibiotic activities and display the inhibition of FtsZ activity [71]. Hemaiswarya et al. reported eight phenylpropanoids against *E. coli* FtsZ, demonstrating them as the inhibitors of FtsZ polymerization and GTPase activity. The light scattering technique showed a dose-dependent reduction in the FtsZ polymerization [72]. Phenylacrylamide possesses antibacterial activity against *Streptococcus pyogenes* and *S. aureus* and prevents their cell division [73], and Vanillin derivatives 3a and 4u were individually tested against *M. tuberculosis* FtsZ [74,75]. Scopoletin, an analog of coumarin related to quercetin and esculetin, inhibits *B. subtilis* FtsZ polymerization and its GTPase activity [76]. The cinnamaldehyde reduces *E. coli* FtsZ polymerization, inhibits its GTPase activity, and is non-lethal to red blood cells [77].

Curcumin is a natural compound extracted from *Curcuma longa*; for ages, it is known for both being used as a source of color as well as a flavoring agent in India and South Asia and retaining several biological properties, such as anticancer, antioxidant, and antibacterial [78]. It has been explored that curcumin binds to tubulin and has antiproliferative activity by disrupting microtubules [79]. With the help of computational docking program, cavity depth analysis, and molecular electrostatic potential (MEP), Kaur et al. recognized possible curcumin attaching sites in *E. coli* FtsZ and *B. subtilis* FtsZ, further verified through mutagenesis studies [80]. Curcumin raises GTPase activity and disrupts FtsZ polymerization, therefore, decreasing the steady-state length of polymer assembly [81]. Colchicine was extremely active against tubulin polymerization but demonstrated no effect on *M. tuberculosis* FtsZ polymerization [63]. Sulfoalkylresorcinol prevents the GTPase activity of FtsZ protein *in vitro* and shows antimicrobial activity against several bacterial organisms [82]. A synthetic derivative, n-undecyl gallate, was reported to disturb ZapA localization and possibly Z-ring development in *Xanthomonas citri* subsp. *citri* [83]. Totarol is a diterpenoid phenol isolated from *Podocarpus totara* that impedes the growth of many Gram-positive bacteria, including *M. tuberculosis*, which leads to the inhibition of FtsZ polymerization and prevention of its GTPase activity. It causes filamentation in *B. subtilis* and demonstrates a mislocalized Z-ring [84]. Initially, it was described that totarol has no activity against human tubulin; recently, it has been verified that totarol attaches to proteins and inhibits FtsZ by forming clumps [85]. Germacrene D-4-ol and germacrene D extracted from the oil of pine needles belong to the terpenoids family. They show antimicrobial activity toward many bacterial species. A docking model expects a binding position of the germacrene family to be a hydrophobic pocket in the FtsZ protein [86].

### Polyphenols

*In vitro*, a high throughput screening assay was used, examining from a library of over 100,000 extracts of plants and microbial fermented broths, to discover a novel inhibitor of FtsZ; they found viriditoxin as an FtsZ inhibitor. Viriditoxin not only exhibits the inhibition of both FtsZ polymerization and GTPase activity *in vivo* and *in vitro* but also shows broad-spectrum activity against Gram-positive antibiotic-resistant and sensitive strains. Moreover, it is non-toxic to human cells, which makes it a fascinating compound for the uncovering of the drug [65]. Plumbagin is a naphthoquinone derivative of *Plumbago zeylanica*, being produced as a secondary metabolite in the roots [87]. It has been shown that plumbagin prevents FtsZ polymerization in a dose-dependent manner. The computational study revealed that the binding site of plumbagin is close to the C-terminal of *B. subtilis* FtsZ. Although it does not display any effect on the *E. coli* FtsZ, plumbagin obviously hinders the polymerization of *B. subtilis* FtsZ. This designates that there is a considerable variation in the structure of FtsZ proteins from different bacteria [88]. SA-011 was manufactured as an analog of plumbagin and prevented the GTPase activity of *Bacillus anthracis* FtsZ a bit stronger in comparison to berberine [89]. Resveratrol could inhibit Z-ring development and also suppress the FtsZ mRNA expression [90].

Chrysophaentins are polyoxygenated, polyhalogenated, and bisdiarylbutene ether macrocycle isolated from algae *Chrysophaeumtaylori*. Plaza et al. suggested that chrysophaentins inhibit both the GTPase activity and the polymerization of FtsZ, which results in activation against various bacteria strains such as *Enterococcus faecium* and *S. aureus* [91]. *In vitro* and *in vivo*, the compound was explored to be a competitive inhibitor of the FtsZ by attaching to the GTP binding site of the protein. Molecular docking experiments demonstrated that the chrysophaentins block a significant part of the GTP binding site of the proteins [91,92]. Five polyphenols known as Zantrins have been recognized. These compounds inhibit the GTPase activity of *E. coli* FtsZ [93], prevent the FtsZ polymerization, and disrupt *E. coli* Z-ring assembly. Zantrins have broad-spectrum antibacterial activity against both Gram-positive and Gram-negative pathogenic bacteria. Two more polyphenols extracted from natural sources have a parallel structure of Zantrin Z1, and likewise, the same antibacterial activity [94] (summary of the FtsZ targeting compounds is listed in Table 1).

**TABLE 1:**
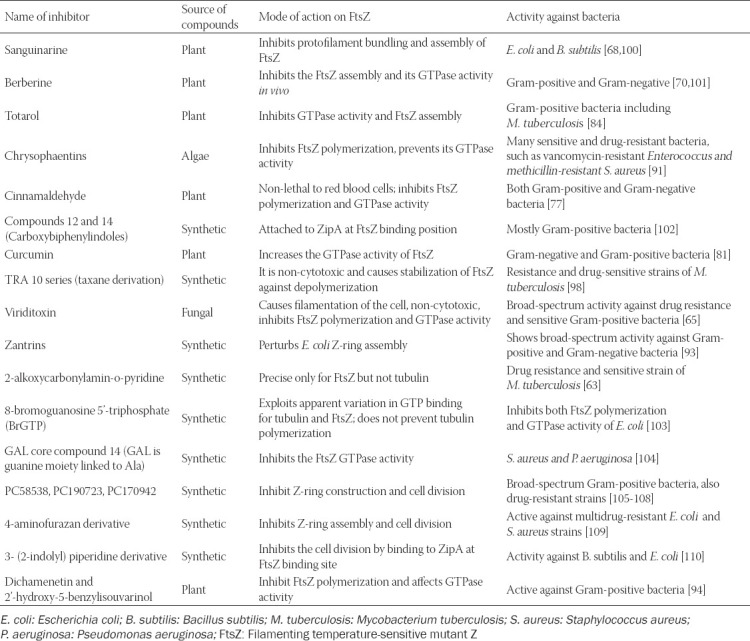
Summary of the cell division protein inhibitors

### Taxane derivative

Paclitaxel, a stabilizing agent of the microtubule that is originally isolated from the bark of the Pacific yew tree, is recognized as a very significant cancer chemotherapy drug [95]. To treat human tumors by targeting tubulin, the taxane derivative taxol (paclitaxel) was used [96]. About 120 taxane derivatives and many compounds with antituberculosis activities were recognized. Optimization of the particular compounds led to the detection of the taxane group, C-seco-taxane multidrug-resistant reversal agents (C-seco-TRAs): they are non-cytotoxic but active against both drug-resistant and sensitive strain of *M. tuberculosis*, having MIC_99_ values in the micromolar range [97,98]. Recently, for the inhibition of the vital mycobacterium FtsZ, a diverse range of commercial analogs and a novel series of sulindac derivatives were screened. Demonstrating studies recommend that these analogs attach to a precise area of *M. tuberculosis* FtsZ polymer which differs from eukaryotic tubulin [99]. However, due to the low structural similarity and sequence identity between the FtsZ and tubulin, recognition for this exact class of compound for *M. tuberculosis* FtsZ may be recommended.

## CONCLUSION

Several bacterial cell division proteins with similar or novel functions have recently been explored to possess a key role in antibiotic discovery. The inhibition of their functions and ability to assemble as part of the division machinery consequently leads to the loss of viability in several bacterial species. Although the core cell division proteins are the same in most bacterial species examined thus far, various additional proteins exist that are sole to their genus; in this way, their inhibition may contribute to a species-specific antimicrobial agent. Our knowledge of the bacterial cell division proteins has recently prompted the exploration of a major player, FtsZ, as a novel antibacterial drug target, but this is still not enough. Therefore, novel or known antibacterial drug targets need to be further elucidated for the development of antibiotics.
